# Conformational effects on iodide binding: a comparative study of flexible and rigid carbazole macrocyclic analogs

**DOI:** 10.3762/bjoc.21.181

**Published:** 2025-11-03

**Authors:** Guang-Wei Zhang, Yong Zhang, Le Shi, Chuang Gao, Hong-Yu Li, Lei Xue

**Affiliations:** 1 State Key Laboratory of Flexible Electronics (LoFE) & Institute of Advanced Materials (IAM), Nanjing University of Posts & Telecommunications, 9 Wenyuan Road, Nanjing 210023, China

**Keywords:** anion recognition, carbazole macrocycles, conformational selection, induced-fit, supramolecular chemistry

## Abstract

To our knowledge, this work represents one of the earliest comparative studies on the anion-binding behaviors of carbazole-based structural analogs, demonstrating that a flexible macrocycle markedly improves iodide binding affinity via an induced-fit mechanism. The flexible analog **PBG** exhibits a 22.78-fold higher fluorescence quenching efficiency upon iodide binding compared to the rigid **WDG** (*K***_PBG_**/*K***_WDG_** = 22.78), demonstrating its potential as a highly sensitive optical probe and offering a novel strategy for engineering dynamic supramolecular receptors. Two carbazole-based macrocyclic probes, **PBG** (flexible benzene ring) and **WDG** (rigid fluorene backbone), were synthesized via Friedel–Crafts reactions. Their iodide (I^−^) recognition properties were systematically explored using ^1^H NMR, UV–vis absorption, and fluorescence spectroscopy. Quantitative analysis via the Benesi–Hildebrand equation and nonlinear fitting demonstrated that flexible **PBG** achieves superior I^−^ binding (*K***_PBG_** = 1.387 × 10^5^ M^−1^) through induced-fit conformational adjustments, whereas rigid **WDG** (*K***_WDG_** = 6.089 × 10^3^ M^−1^) is constrained by preorganized cavity geometry, adhering to a conformational selection mechanism. This work elucidates the synergistic interplay between conformational dynamics and localized structural adaptations governing anion recognition. The findings advance the rational design of tunable, high-affinity anion receptors and deepen the understanding of conformational regulation in supramolecular systems.

## Introduction

Macrocyclic compounds have garnered significant attention in supramolecular chemistry and anion recognition owing to their tunable cavity geometries and binding capabilities [1–8]. Notably, the anion-binding performance of these macrocycles is inherently governed by their conformational dynamics [9–10]. Flexible hosts adaptively adjust their cavities via induced-fit mechanisms to accommodate diverse guests, while rigid hosts rely on preorganized spatial complementarity for selective recognition. Balancing binding affinity and selectivity through precise conformational engineering remains a critical challenge [11]. Previous studies, such as those by Tian et al. [12], demonstrated selective binding in chiral assemblies via crown ether chain-length modulation. The dynamic interplay between conformational selection and induced-fit mechanisms in isomeric systems remains underexplored. Cram’s preorganization theory [13] posits that prestructured hosts with minimized solvation exhibit enhanced binding stability. Recent advances [14–16] highlight the pivotal role of conformational dynamics, prompting a paradigm shift from static binding models to mechanistic insights into induced fit [17] and conformational selection [18]. This cognitive shift marks the transition of molecular recognition research from a simple description of binding phenomena to a deep understanding at the molecular level of the dynamic regulation mechanisms of conformations [19–20].

Although the influence of conformational dynamics on molecular recognition has been paid attention to, there is still a gap in the comparative study of flexible (**PBG**) and rigid (**WDG**) structural analogs of the same framework. In order to solve the above problems, in this study, carbazole macrocyclics were used as models [21–22], and the structural analogs of phenyl group (**PBG**, flexible) and fluorene group (**WDG**, rigid) were introduced by Friedel–Crafts reaction. It should be emphasized that since the two single bonds in **WDG** are rotationally restricted, all its accessible conformations constitute a subset of **PBG**'s conformations. The binding constants and conformational change mechanisms of the two analogs were quantitatively evaluated by proton nuclear magnetic resonance spectroscopy (^1^H NMR), ultraviolet–visible absorption spectroscopy and fluorescence spectroscopy, combined with Benesi–Hildebrand equation [23] and nonlinear curve fitting [24]. The purpose of systematically exploring the difference between the two types of recognition of I^−^ is to reveal the regulation law of conformational dynamics on the binding mechanism.

## Experimental

**Scheme 1:**
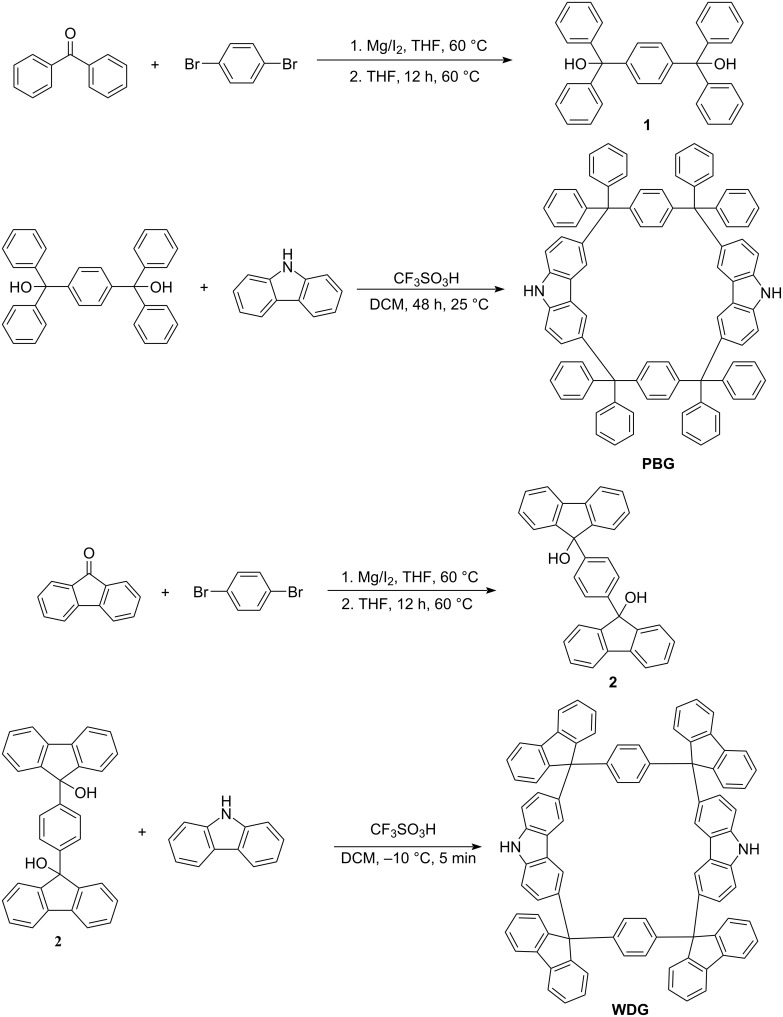
Synthesis routes of **PBG** and **WDG**.

**PBG (**CCDC Number: 2070280**)** and **WDG** were synthesized from phenyl- and fluorenyl-substituted precursors, respectively, via Friedel–Crafts alkylation (Scheme 1) according to our previous work [22]. Structural characterization was performed using ^1^H NMR (Figures S1 and S2) and mass spectrometry (Figure S3) in Supporting Information File 1. **WDG** exhibited poor solubility, which hindered the direct acquisition of its single crystal structure for characterization. However, after Boc (*tert*-butyloxycarbonyl) protection, a single crystal structure (CCDC Number: 2339028) of the modified compound was successfully obtained. This indirectly confirmed the molecular structure of **WDG** [22].

**Figure 1:**
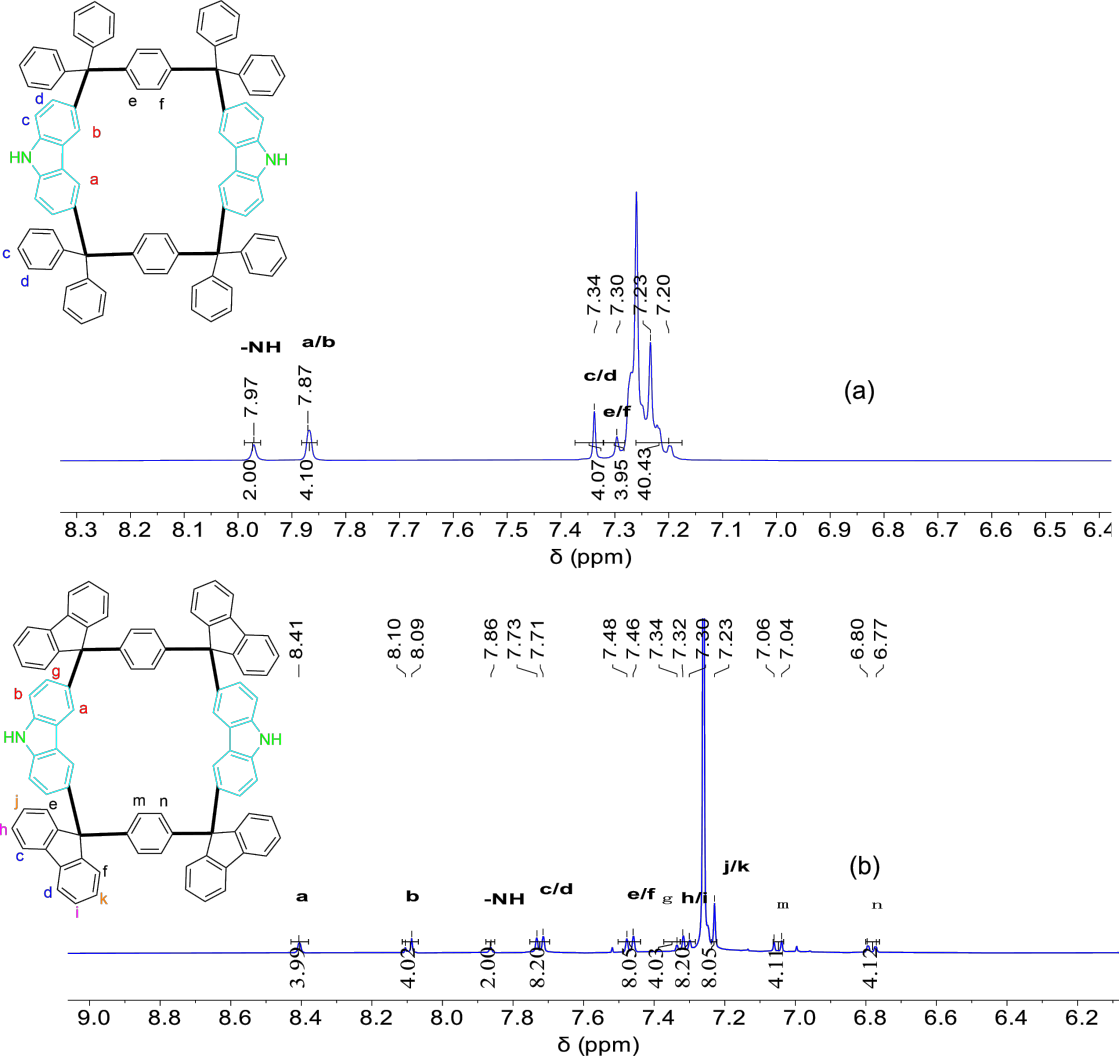
(a) partial ^1^H NMR spectrum of **PBG** in CDCl_3_ (400 MHz, CDCl_3_, 25 °C), (b) Partial ^1^H NMR spectrum of **WDG** in CDCl_3_ (400 MHz, CDCl_3_, 25 °C).

The NH protons of the carbazole moiety in **PBG** and **WDG** were observed as singlets at δ 7.9706 and δ 7.8624, respectively, in their ^1^H NMR spectra (Figure 1a and 1b).

In order to study the role and mechanism of anion recognition by the two structural analogs, we added a commercially available tetrabutylammonium salt (TBAI) to the body dissolved in CDCl_3_ (TBAI has a good solubility in CDCl_3_) and studied the interaction between the receptor and iodide ion by ^1^H NMR titration, UV–vis absorption spectroscopy and fluorescence spectroscopy.

## Results and Discussion

**Figure 2:**
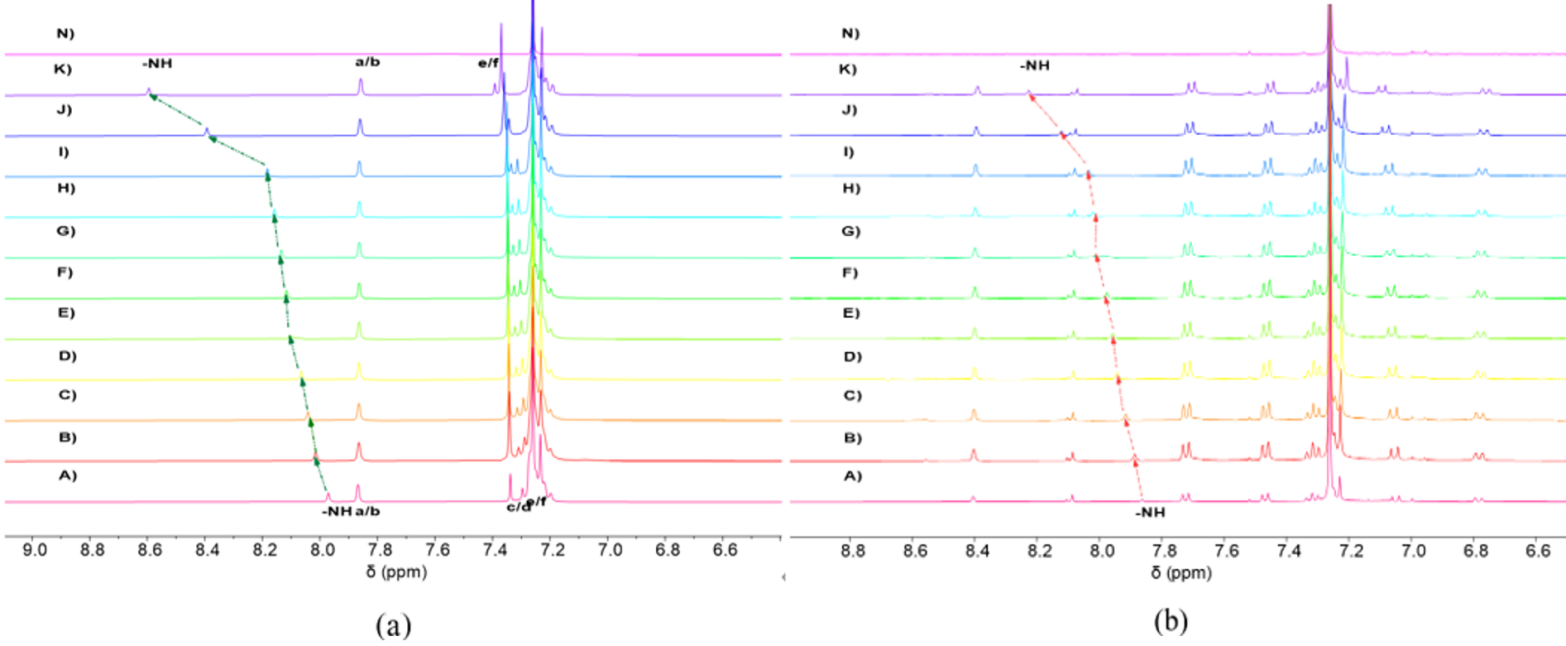
(a) Partial ^1^H NMR spectra of **PBG** and TBAI at different equivalent concentrations in CDCl_3_ (400 MHz, CDCl_3_, 25 °C); (A) **PBG**, (N) TBAI, and equimolar TBAI solutions at concentrations of (B) 0.25, (C) 0.50, (D) 0.75, (E) 1.00, (F) 1.25, (G) 1.5, (H) 1.75, (I) 2.00, (J) 5.00, (K) 10.00 equivalents, (b) Partial ^1^H NMR spectra of **WDG** and TBAI at different equivalent concentrations in CDCl_3_ (400 MHz, CDCl_3_, 25 °C); (A) **WDG**, (N) TBAI, and equimolar TBAI solutions at concentrations of (B) 0.25, (C) 0.50, (D) 0.75, (E) 1.00, (F) 1.25, (G) 1.5, (H) 1.75, (I) 2.00, (J) 3.00, (K) 5.00 equivalents.

Partial ^1^H NMR spectra of **PBG** and **WDG** after binding to tetrabutylammonium iodide (TBAI) are presented in Figure 2a and 2b, respectively. All ^1^H NMR titrations were conducted at a fixed concentration of 8.7 mM. With the addition of 2 equivalents TBAI, the NH peak on the **PBG** shifts down by 0.1902 ppm. When the concentration was increased to 5 equivalents, the NH signal peak of carbazole continued to move towards downfield, with a displacement of 0.4004 ppm to 8.3929 ppm. When the concentration was increased to 10 equivalents, the NH signal peak of carbazole continued to move towards downfield, with a displacement of 0.6015 ppm to 8.5940 ppm. This phenomenon may be due to the formation of noncovalent bonds between the iodine ions encapsulated inside the macrocyclic and the hydrogen atoms on the macrocyclic framework, which reduces the density of the electron cloud of aromatic groups inside the macrocycle and thus causes the NH peak to be shifted downfield. At the same time, after the addition of TBAI to the **PBG** solution, both proton signals c/d and e/f were shifted downfield, while protons a/b moved slightly to higher field, confirming the interaction between the anion and the acceptor. For **WDG**, the carbazole NH peak on **WDG** was shifted 0.1725 ppm downfield when 2 equivalents TBAI was added. When the concentration increased to 5 equivalents, the NH signal peak of carbazole continued to move downfield, with a displacement of 0.3649 ppm to 8.2273 ppm, while proton signals a/b and c/d both moved slightly to higher field. This indicates that there is a slight difference in the interaction between NH protons and iodine ions of the two structural analogs. Structural analysis showed that since the two single bonds are rotationally restricted, all its accessible conformations of **WDG** constitute a subset of **PBG**'s conformations. Consequently, **PBG** exhibits greater conformational diversity and enhanced flexibility compared to **WDG**, which explained its higher binding capacity. The conformational dynamics of flexible **PBG**s allow them to adapt to I^−^ through the induced-fit mechanism, while rigid **WDG**s are limited by the preorganization effect and can only achieve weak hydrogen bonding through conformational selection. In addition, the changes in the chemical shifts of the above two structures lead us to believe that the two structural analogs undergo conformational changes when interacting with iodine ions, and that flexible **PBG**s enhance the host–guest complementarity through conformational adjustments, resulting in local conformational fitting changes [25].

To determine the binding ratios and binding constants of the two structural analogs to iodine ions, we designed a Job plot obtained by varying the ratio of host and guest, but the total concentration of host and guest is fixed: [host] + [guest] = 5.0 mM, plotting the Job plot obtained by the chemical shift change of the host NH proton in the ^1^H NMR spectrum, see Figures S4 and S5 in Supporting Information File 1. This experiment supports a 1:1 stoichiometric binding between the guest TBAI and the host **PBG** in CDCl_3_.

**Figure 3:**
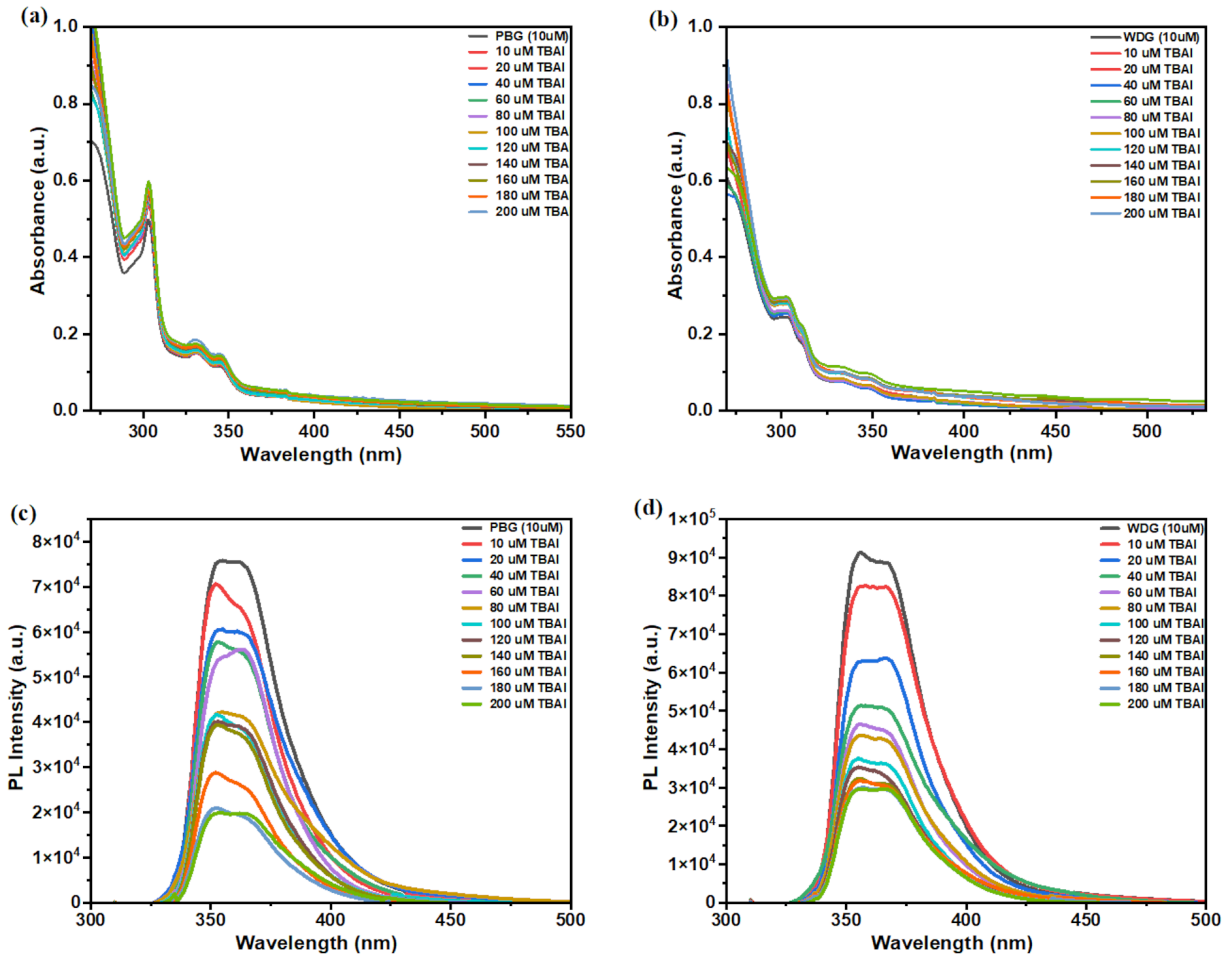
(a) UV–vis spectra of **PBG** (10 μM) in CHCl_3_ with TBAI concentration, (b) UV–vis spectra of **WDG** (10 μM) in CHCl_3_, (c) Fluorescence spectra of **PBG** (10 μM) in CHCl_3_ (λ_ex_: 303 nm), (d) Fluorescence spectra of **WDG** (10 μM) in CHCl_3_ (λ_ex_: 303 nm).

Then, the binding affinity of the two receptors to iodine ions was further studied, and the changes of UV–visible absorption and fluorescence spectra of the two receptors to anions in CHCl_3_ were monitored during titration with TBAI concentrations with different equivalent ratios. As shown in Figure 3a and 3b, the UV absorption bands of both **PBG** and **WDG** are significantly enhanced when I^−^ is continuously added, indicating that there is an efficient bond between the two receptors and I^−^.

According to the linear expression of Benesi–Hildebrand, the variation of [1/(A-A_0_)] and 1/[I^−^] measured at 303 nm is linear (*R^2^***_PBG_** = 0.99625, *R^2^***_WDG_** = 0.99464) [26], see Figures S12 and S13 in Supporting Information File 1, which indicates the formation of a 1:1 complex between the two structural analogs and iodine ions, which is consistent with the conclusions reached by our NMR titration. The binding constants of the complexes (*K*), *K***_PBG_** = (1.387 ± 0.02363) × 10^5^ M^−1^, *K***_WDG_**= (6.089 ± 0.3320) × 10^3^ M^−1^ were measured (Tables S1 and S2 in Supporting Information File 1), indicating that both acceptors had strong binding ability to iodide ions in chloroform solution. It should be noted that the titration curve did not approach saturation, which may be attributed to conformational changes occurring during the quenching process. At high quencher concentrations, structural alterations in fluorophores could induce variations in fluorescence properties, leading to deviations from the linear Stern–Volmer relationship governing the quenching mechanism. The flexible benzene ring of the **PBG** allows the cavity to be conformationally adjusted to fit the size of I^−^, while the rigid fluorenyl group of the **WDG** results in steric hindrance, with a 95.6% lower binding constant of *K***_WDG_**/*K***_PBG_** = 0.044, a difference that can be attributed to the synergistic effect of induced fit (**PBG**) and conformational selection (**WDG**). Fluorescence emission spectra further corroborated the response of **PBG** and **WDG** to iodide ions (Figure 3c and 3d). When TBAI is added to two different conformations of the receptor molecule, the fluorescence emission intensity decreases. However, during the whole titration of anion fluorescence spectroscopy, only the fluorescence emission intensity changed, no peak position changed, and no new fluorescence emission peaks appeared, indicating that photoelectron transfer occurred between iodine ions and the macrocycles [27–28], and fluorescence quenching was due to the photoinduced electron transfer (PET) effect between iodine ions and acceptors, resulting in non-radiative dissipation of excited state energy rather than the formation of new excited state complexes. As the I^−^ content continued to increase, the fluorescence emission intensity of **PBG** continued to decrease at 354 nm and 364 nm. Similarly, the fluorescence emission intensity of **WDG** at 356 nm and 369 nm decreases as the I^−^ content increases.

Additionally, as a control experiment, the interaction between carbazole molecules and iodide ions via UV–vis and PL spectroscopy (Figures S14 and S15 in Supporting Information File 1) were investigated. The results revealed no analogous regular trends, and the interactions could not be effectively modeled. This further corroborates the decisive role of the macrocyclic architecture in the anion recognition process.

Quantum chemical calculations were performed with the ORCA 5.0.3 software [29]. The geometry optimization was performed by using B3LYP with the def2-SVP basis set. As illustrated in the computational schematic (Figures S16 and S17 in Supporting Information File 1), the anions are positioned within the macrocycle cavity, exhibiting close contacts to hydrogen atoms on the bridging benzene rings, peripheral substituted phenyl groups, and carbazole moieties. These spatial interactions suggest a synergistic binding mechanism driven by cavity complementarity, and the energy of the **PBG**–iodide complex is lower than that of the **WDG**–iodide complex, it indicates that **PBG** forms a thermodynamically more stable complex with iodide ions compared to **WDG**. This stability difference arises from the distinct conformational behaviors of the two macrocycles. **PBG**'s flexible benzene ring allows for induced-fit binding, where the macrocycle dynamically adjusts its cavity to optimize interactions with iodide, minimizing energy through conformational adaptability. **WDG**'s rigid fluorene backbone relies on a preorganized cavity, which may not perfectly complement iodide's size or geometry, leading to weaker interactions and a higher-energy (less stable) complex. This energy difference aligns with the observed higher binding constant (*K*) of **PBG**, demonstrating that structural flexibility enhances anion-binding efficiency by enabling dynamic host–guest complementarity. Such insights are critical for designing adaptive receptors for applications like environmental sensing or selective ion extraction.

## Conclusion

In summary, two carbazole-based macrocyclic structural analogs (**PBG** and **WDG**) were synthesized via a conformational engineering strategy. Both macrocycles demonstrated distinct iodide (I^−^) recognition capabilities in chloroform and varying degrees of fluorescence quenching, as evidenced by ^1^H NMR titration, UV–vis absorption, and fluorescence spectroscopy. The flexible **PBG** exhibited a 22.78-fold higher binding constant (*K* = 1.387 × 10^5^ M^−^¹) compared to the rigid **WDG** (*K* = 6.089 × 10³ M^−^¹), highlighting the critical role of conformational adaptability in anion recognition. Mechanistic studies revealed that **PBG** may operate through an induced-fit mechanism, dynamically adjusting its cavity to optimize I^−^ binding, whereas **WDG** relies on a preorganized cavity with limited conformational flexibility, adhering to a conformational selection model. This work provides fundamental insights into the interplay between conformational dynamics and supramolecular recognition.

The conformational flexibility of **PBG** enables efficient iodide recognition and suggests possible applications in environmental monitoring, particularly for detecting radioactive iodine species in nuclear waste streams. Additionally, the cavity size of the macrocycle can be precisely modulated by varying the number of bridging benzene substituents, which provides a foundation for developing next-generation smart sensors with programmable anion selectivity.

## Supporting Information

File 1 Additional experimental data.

## Data Availability

All data that supports the findings of this study is available in the published article and/or the supporting information of this article.
