# Supplementary material for: Conformational effects on iodide binding: a comparative study of flexible and rigid carbazole macrocyclic analogs
Source: Beilstein J Org Chem. 2025 Nov 3;21:2369–75. doi: 10.3762/bjoc.21.181 (PMC12599402; doi:10.3762/bjoc.21.181)
Supplement: File 1 — Additional experimental data. [file Beilstein_J_Org_Chem-21-2369-s001.pdf]

BEILSTEIN JOURNAL OF ORGANIC CHEMISTRY

Supporting Information

for

Conformational effects on iodide binding: a comparative study of flexible and rigid carbazole macrocyclic analogs

Guang-Wei Zhang, Yong Zhang, Le Shi, Chuang Gao, Hong-Yu Li and Lei Xue

Beilstein J. Org. Chem. **2025,** *21,* 2369–2375. doi:10.3762/bjoc.21.181

Additional experimental data

License and Terms: This is a supporting information file under the terms of the Creative Commons Attribution License (https://creativecommons.org/licenses/by/4.0). Please note that the reuse, redistribution and reproduction in particular requires that the author(s) and source are credited and that individual graphics may be subject to special legal provisions.
The license is subject to the *Beilstein Journal of Organic Chemistry* terms and conditions: (https://www.beilstein-journals.org/bjoc/terms)

Table of contents

1. General information of synthesis..S2

2. General experimental and synthetic methods for **PBG** and **WDG**......S2

2.1 Compound 1: 1,4-Phenylenebis(diphenylmethanol)...................S2

2.2 Compound 2: 9,9-(1,4-Phenylene)bis(9H-fluoren-9-ol)..............S3

2.3 Compound **PBG**..S4

2.4 Compound **WDG**..S4

3. MALDI-TOF-MS analysis of **PBG** and **WDG**..................................S5

4. Job's plot and determination of Binding Constants S7

5. Binding Constants Determined by UV–vis titrations........................S11

5.1 Binding constants for **PBG**..S11

5.2 Binding constants for **WDG**..S12

6. Quantum chemical calculations..S13

7. References S24

1. General information of synthesis

Chloroform (CH_3Cl) was dried over anhydrous sodium sulfate and distilled under reduced pressure. All reagents were purchased from commercial suppliers and used without further purification. Nuclear magnetic resonance (NMR) spectra were recorded on a Bruker 400 MHz NMR spectrometer ($CDCl_3$ as solvent, TMS as internal standard; chemical shifts reported in ppm). Molecular weights were determined using a Shimadzu 2010 PLUS mass spectrometer. UV–vis absorption spectra were acquired on a LAMBDA 35 spectrophotometer, and fluorescence spectra were measured using an RF-6000 Plus fluorescence spectrophotometer.

2. General experimental and synthetic methods for PBG and WDG

2.1 Compound 1: 1,4-Phenylenebis(diphenylmethanol)

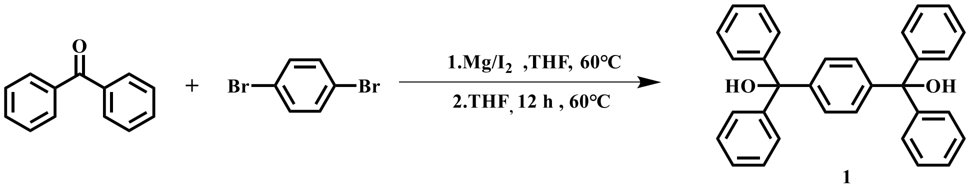

1

All glassware was dried prior to use. Magnesium chips (0.31 g, 12.8 mmol) and catalytic iodine were added to a 100 mL three-necked flask under nitrogen. A solution of 1,4-dibromobenzene (1.0 g, 4.2 mmol) in anhydrous tetrahydrofuran (THF, 40 mL) was added dropwise to the flask. The mixture was heated until the solution turned transparent, then stirred at 60 °C for 4 h to form the Grignard reagent. This reagent was transferred to a solution of benzophenone (2.33 g, 12.8 mmol) and refluxed at 60 °C for 12 h. The reaction was quenched with saturated NH_4Cl solution, extracted with CH_2Cl_2, and purified by column chromatography (petroleum ether/ethyl acetate = 5:1, v/v) to yield 1,4-phenylenebis(diphenylmethanol) as a white solid (1.53 g, 82%). **^{1}H NMR (400 MHz, $CDCl_3$):** δ 7.37 (d, J = 2.2 Hz, 2H), 7.36 (s, 4H), 7.34 (d, J = 4.0 Hz, 14H), 7.31 (d, J = 3.8 Hz, 4H), 2.83 (s, 2H, J = 2.2 Hz).

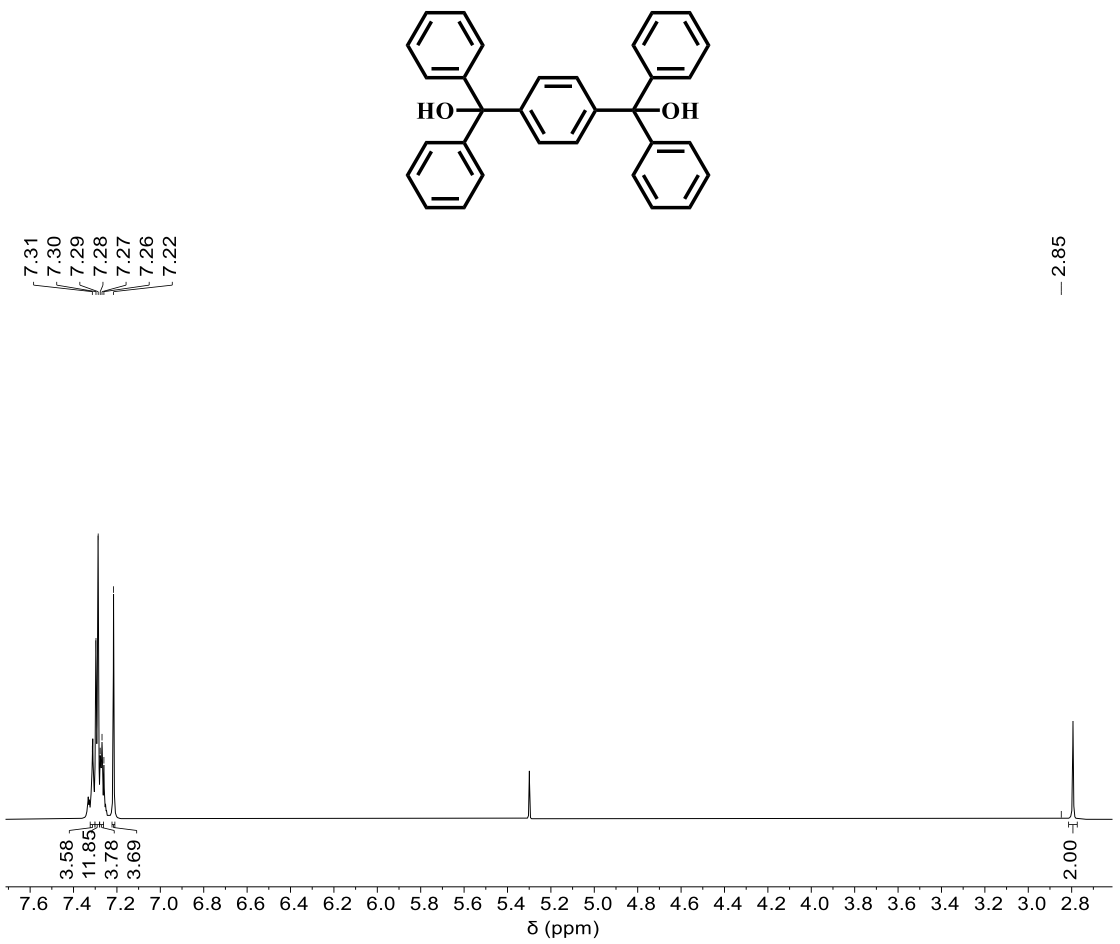

Figure S1 ^{1}H NMR spectrum (400 MHz, $CDCl_3$, 25℃) of compound **1**.

2.2 Compound 2: 9,9‘-(1,4-Phenylene)bis(9*H*-fluoren-9-ol)

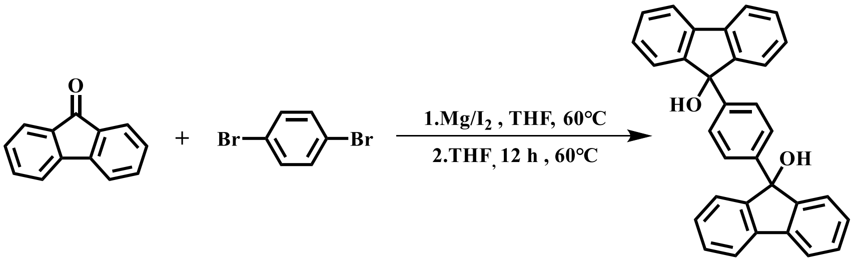

2

The intermediate 9,9‘-(1,4-Phenylene)bis(9*H*-fluoren-9-ol) (yellow solid, yield 68%) was prepared by replacing benzophenone with 9-fluorenone (2.8 g, 15.0 mmol) according to the synthesis method of 1,4-phenylenebis(diphenylmethanol). **^{1}H NMR (400 MHz, $CDCl_3$)** δ 7.63 (d, J = 7.5 Hz, 4H), 7.35 (d, J = 1.4 Hz, 1H), 7.33 (d, J = 1.4 Hz, 2H), 7.30 (d, J = 7.9 Hz, 5H), 7.26 – 7.23 (m, 5H), 7.21 (d, J = 7.4 Hz, 3H), 2.38 (s, 2H).

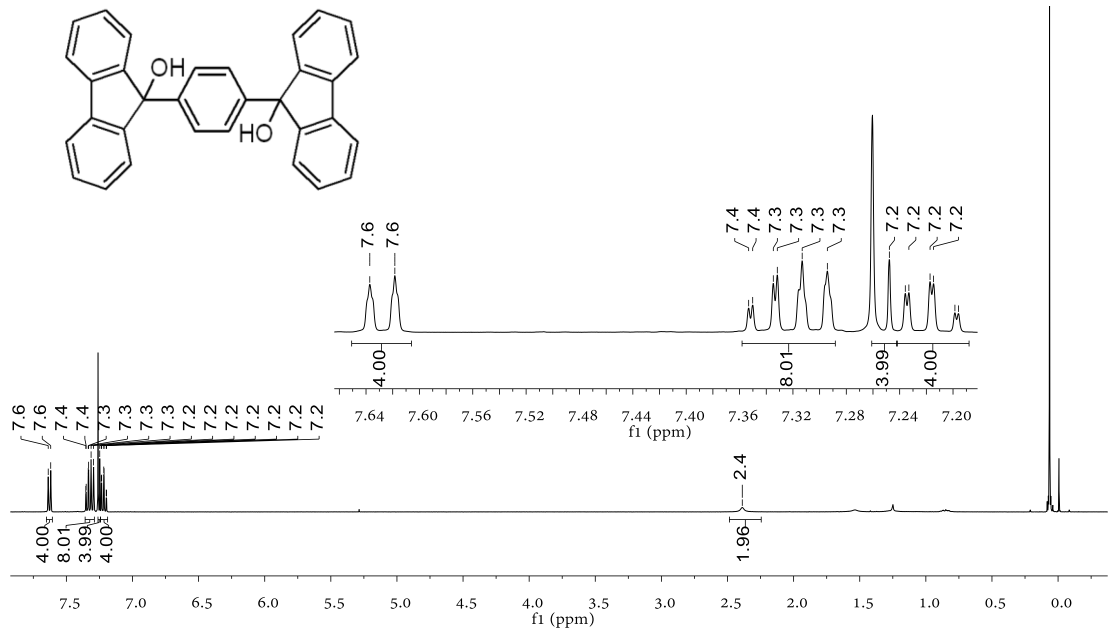

Figure S2 ^{1}H NMR spectrum (400 MHz, $CDCl_3$, 25 °C) of compound **2**.

2.3 Compound PBG

A mixture of **1** (400 mg, 0.91 mmol), carbazole (151.13 mg, 0.91 mmol), and trifluoromethanesulfonic acid (0.2 mL) in CH_2Cl_2 (110 mL) was stirred at 25 ℃ for 48 h. The crude product was purified by column chromatography (CH_2Cl_2/petroleum ether = 1:1, v/v) and recrystallized from ethyl acetate to yield **PBG** (331.87 mg, 32%). **MALDI-TOF-MS (*m/z*):** Calcd for $C_{88}H_{62}N_2$: 1146.49 [M^+]; Found: 1146.837. **^{1}H NMR (400 MHz, $CDCl_3$):** δ 7.97 (s, 2H), 7.87 (s, 4H), 7.34 (s, 4H), 7.30 (s, 4H), 7.28 (s, 8H), 7.25 (d, 38H), 7.20 (d, *J* = 2.0 Hz, 2H). CCDC Number: 2070280.

2.4 Compound WDG [1]

A solution of carbazole (38.1 mg, 0.228 mmol), **2** (100 mg, 0.228 mmol), and trifluoromethanesulfonic acid (0.2 mL) in CH_2Cl_2 (76 mL) was stirred at −10 ℃ for 5 min. The product was precipitated by cold CH_2Cl_2, washed, and dried to yield **WDG** (116.1 mg, 45%). **MALDI-TOF-MS (*m/z*):** Calcd for $C_{88}H_{54}N_2$: 1138.43 [M^+]; Found: 1138.781. **^{1}H NMR (400 MHz, $CDCl_3$):** δ 8.41 (s, 4H), 8.10 (d, *J* = 7.1 Hz, 4H), 7.86 (s, 2H), 7.72 (d, *J* = 6.9 Hz, 8H), 7.47 (d, *J* = 7.1 Hz, 8H), 7.34 (s, 4H), 7.31 (d, *J* = 7.4 Hz, 8H), 7.23 (s, 8H), 7.05 (d, *J* = 8.6 Hz, 4H), 6.78 (d, *J* = 10.4 Hz, 4H).

3. MALDI-TOF-MS analysis of PBG and WDG (matrix: α-cyano-4-hydroxycinnamic acid, laser energy: 30%)

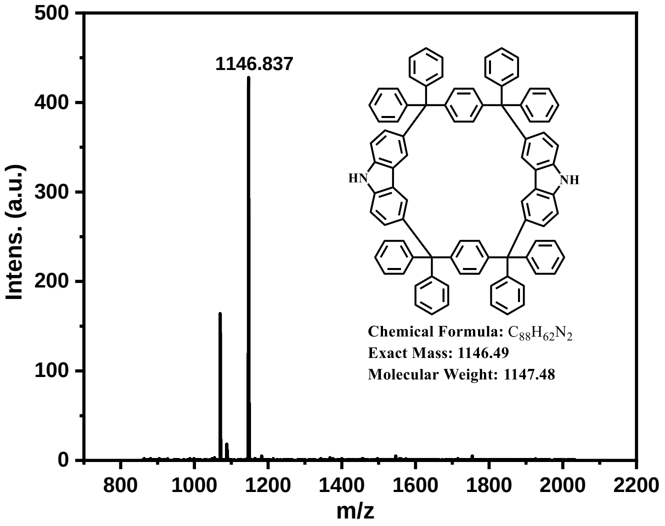

Figure S3 (a) Molecular ion peaks of **PBG**.

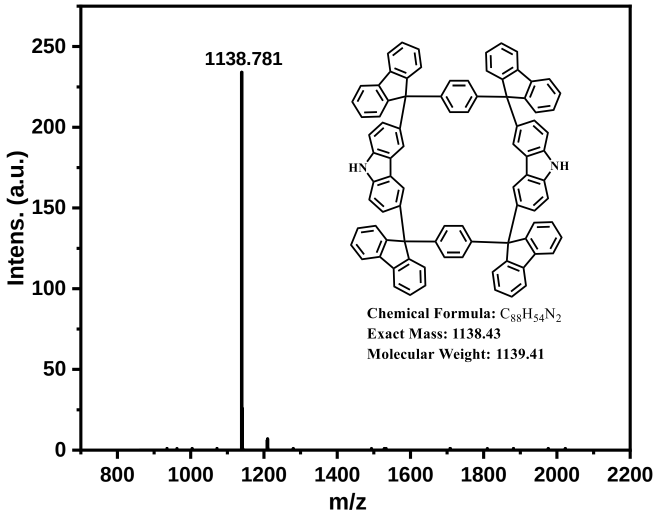

Figure S3 (b) Molecular ion peaks of **WDG**.

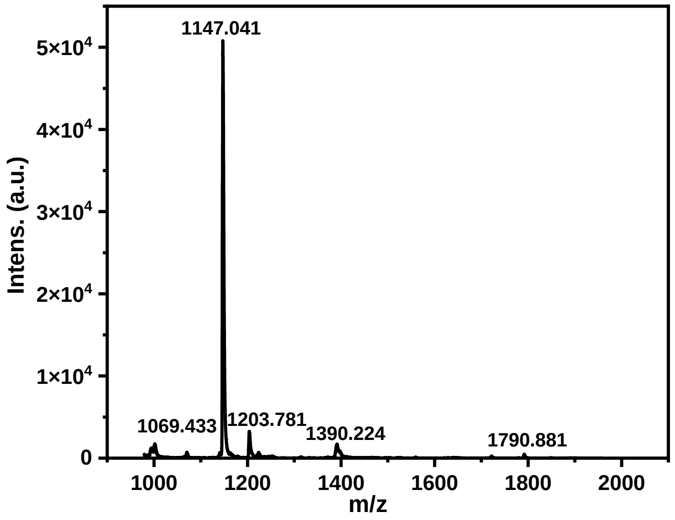

Figure S3 (c) Molecular ion peaks of **PBG** and **TBAI** in negative-ion mode.

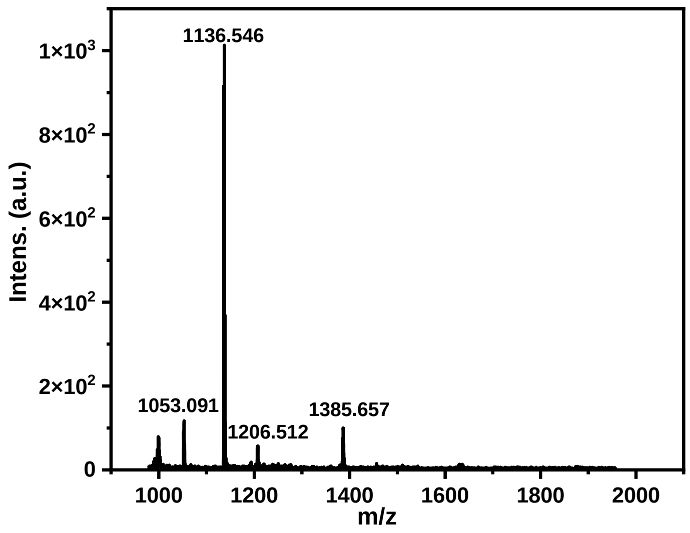

Figure S3 (d) Molecular ion peaks of **WDG** and **TBAI** in negative-ion mode.

4. Job's plot and determination of binding constants

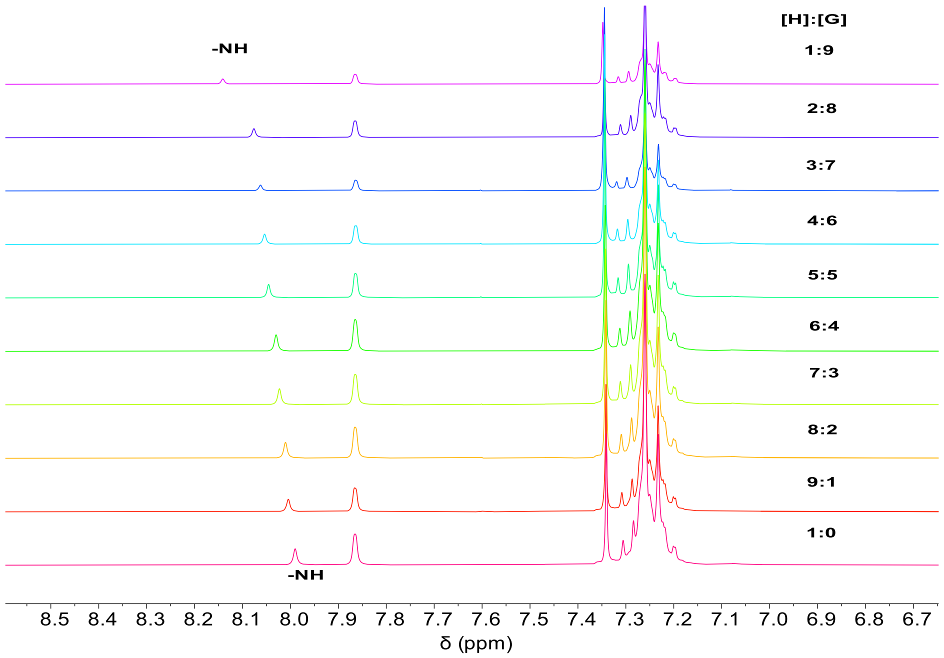

Figure S4 Partial ^{1}H NMR spectra (400 MHz, 25℃, $CDCl_3$) of **PBG** with varying molar ratios of TBAI.

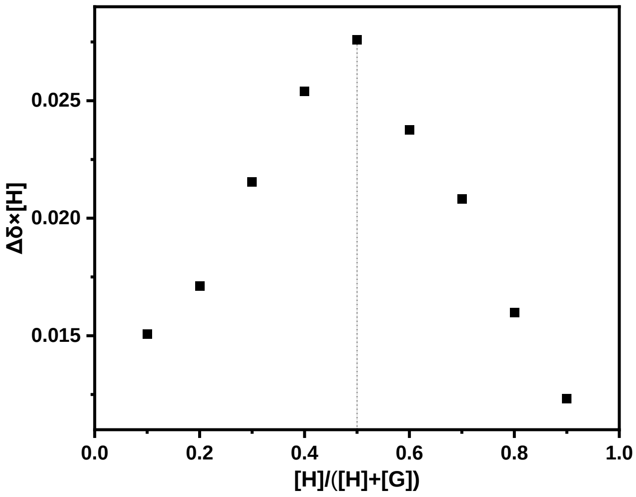

Figure S5 Job's plot obtained by plotting the chemical shift change (Δδ) of the Host's proton NH in ^{1}H NMR spectra by varying the ratio of the host and the tetrabutylammonium iodide (TBAI) against the mole fraction of Host **PBG** The total concentration of the host and the guest is fixed: [Host] + [Guest] = 5.0 mM. The total concentration was selected at 5.0 mM to ensure that the chemical shift change was within the detection limit This experiment supports the 1:1 binding stoichiometry between the TBAI and **PBG** in $CDCl_3$

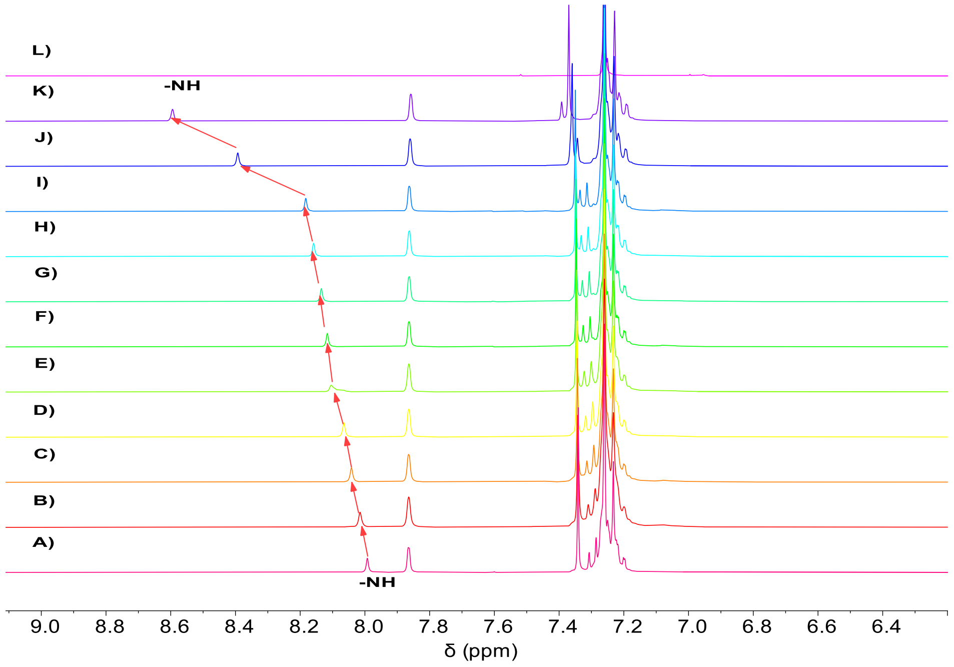

Figure S6 Partial ^{1}H NMR spectra of **PBG** and TBAI at different equivalent concentrations in $CDCl_3$ (400 MHz, $CDCl_3$, 25 ℃); (A) **PBG**, (L) TBAI, and equimolar TBAI solutions at concentrations of (B) 0.25, (C) 0.50, (D) 0.75, (E) 1.00, (F) 1.25, (G) 1.5, (H) 1.75, (I) 2.00, (J) 5.00, (K) 10.00 equivalents.

Nonlinear curve-fitting method was then used to obtain the association constant through the following equation[2]:

$$\delta=\delta_0+\Delta\delta\ (0.5/[H]_0)\ ([G]+[H]_0+1/K-(([G]+[H]_0+1/K)\text{^}2-4[H]_0[G])\text{^}0.5)$$

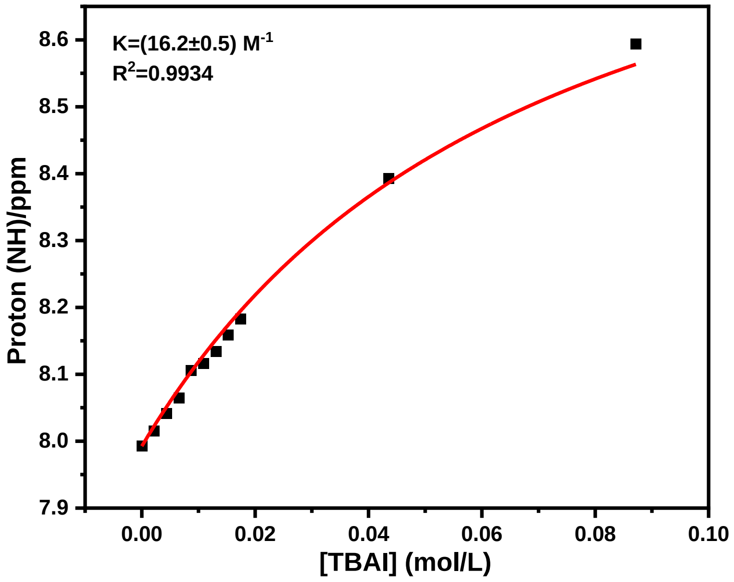

Figure S7 Nonlinear curve fitting based on 1:1 binding model for **PBG**-TBAI (400 MHz, 25 ℃, $CDCl_3$).

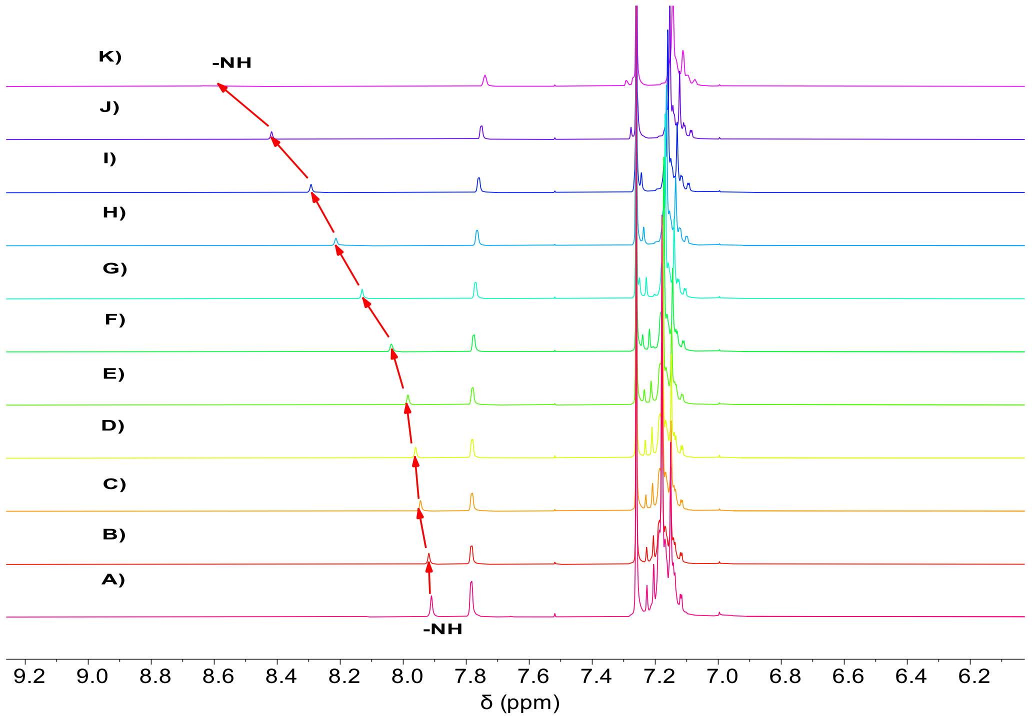

Figure S8 Partial ^{1}H NMR spectra of **PBG** and TBAI at different equivalent concentrations in $CDCl_3$ (400 MHz, $CDCl_3$, 25 ℃); (A) **PBG**, (L)TBAI, and equimolar TBAI solutions at concentrations of (B) 0.5, (C) 1.0, (D) 2.0, (E) 3.0, (F) 5.0, (G) 10.0, (H) 20.0, (I) 30.0, (J) 50.0, (K) 100.0 equivalents.

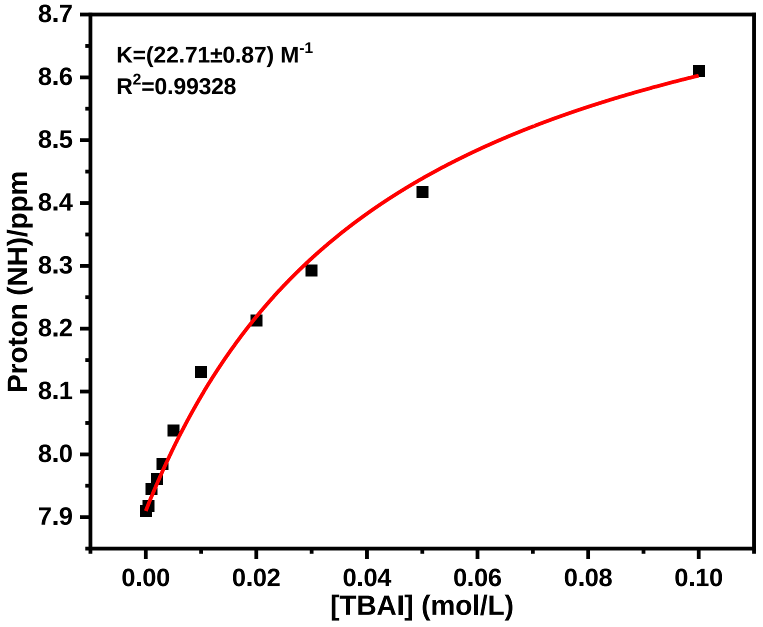

Figure S9 Nonlinear curve fitting based on 1:1 binding model for **PBG**-TBAI (400 MHz, 25℃, $CDCl_3$).

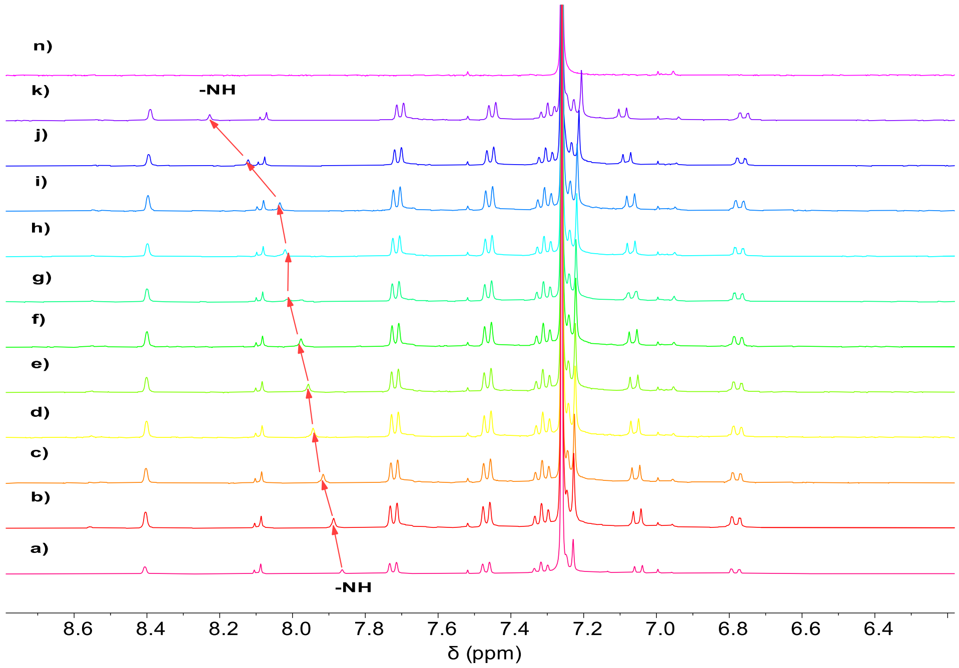

Figure S10 Partial ^{1}H NMR spectra of **WDG** and TBAI at different equivalent concentrations in $CDCl_3$ (400 MHz, $CDCl_3$, 25 ℃); (a) **WDG**, (n) TBAI, and equimolar TBAI solutions at concentrations of (b) 0.25, (c) 0.50, (d) 0.75, (e) 1.00, (f) 1.25, (g)1.5, (h) 1.75, (i) 2.00, (j) 3.00, (k) 5.00 equivalents.

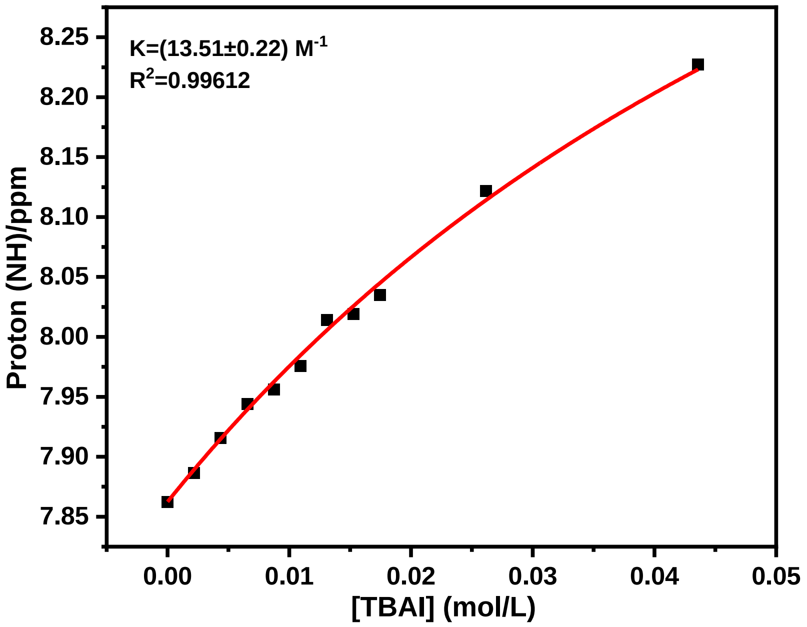

Figure S11 Nonlinear curve fitting based on 1:1 binding model for **WDG**-TBAI (400 MHz, 25 ℃, $CDCl_3$).

5. Binding constants determined by UV–vis titrations[3]

Upon addition of incremental amounts of anions to a $CHCl_3$ solution of **PBG/WDG**, changes in the absorbance features of **PBG/WDG** (10 μM was used, unless otherwise stated) were seen.

5.1 Binding constants for PBG

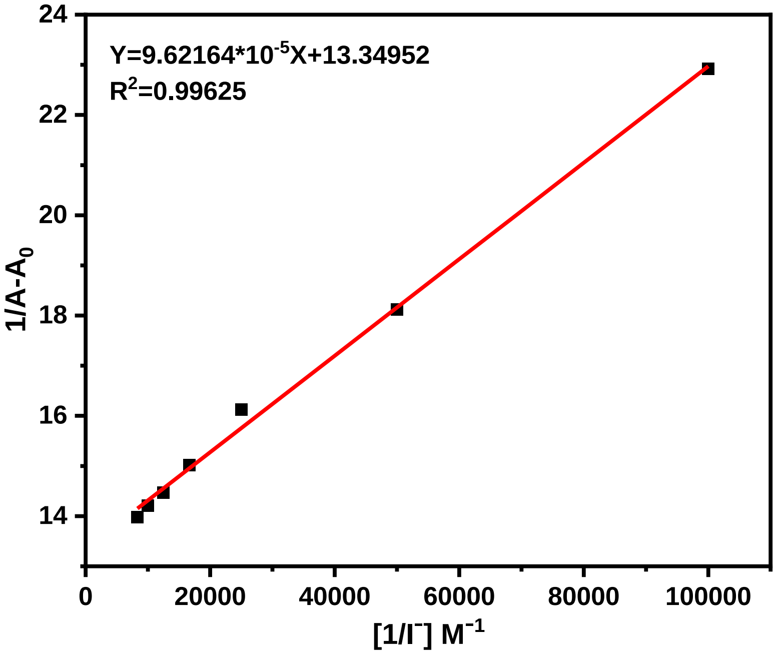

Figure S12 Benesi–Hildebrand plot for **PBG** with TBAI.

Table S1 Association constant (K) of **PBG** with TBAI: $(1.387 \pm 0.0236) \times 10^5$ M^{-1}

Y=A+BX		
coefficient	value	error
A	13.34952	0.10666
B	$9.62164E^{-5}$	$2.40854E^{-6}$
$K_S = A/B = 1.387 \times 10^5$		
$\triangle K_S = \lvert \triangle A/A - \triangle B/B \rvert \times K_S = 2.363 \times 10^3$		
$K = K_S + \triangle K_S = (1.387 \pm 0.02363) \times 10^5\ M^{-1}$		

5.2 Binding constants for WDG

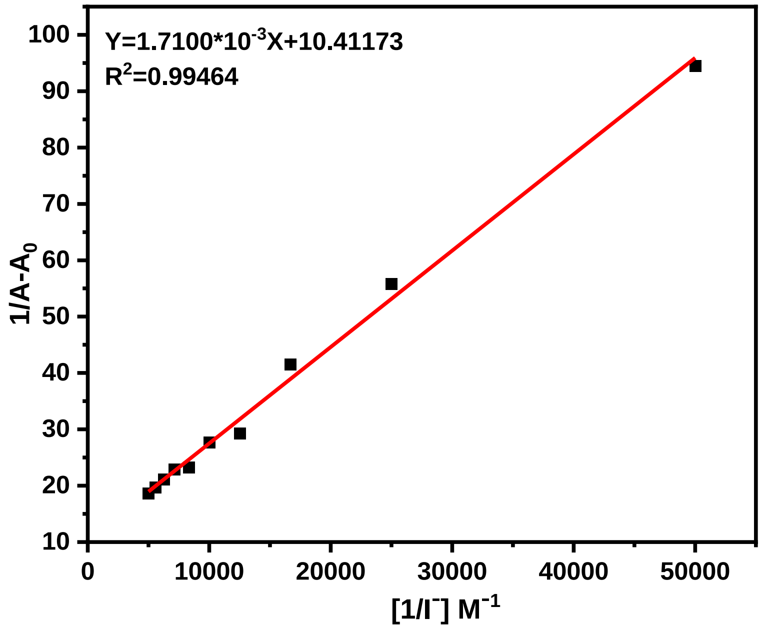

Figure S13 Benesi–Hildebrand plot for **WDG** with TBAI.

Table S2 Association constant (*K*) of **WDG** with TBAI: $(3.069 \pm 0.163) \times 10^3$ M^{-1}.

Y=A+BX		
coefficient	value	error
A	10.41173	0.82332
B	$1.71E^{-3}$	$4.18277E^{-5}$
K_S=A/B=6.089×10^3		
$\triangle K_S = \lvert \triangle A/A - \triangle B/B \rvert \times K_S = 0.3320 \times 10^3$		
$K = K_S + \triangle K_S = (6.089 \pm 0.3320) \times 10^3$ M^{-1}		

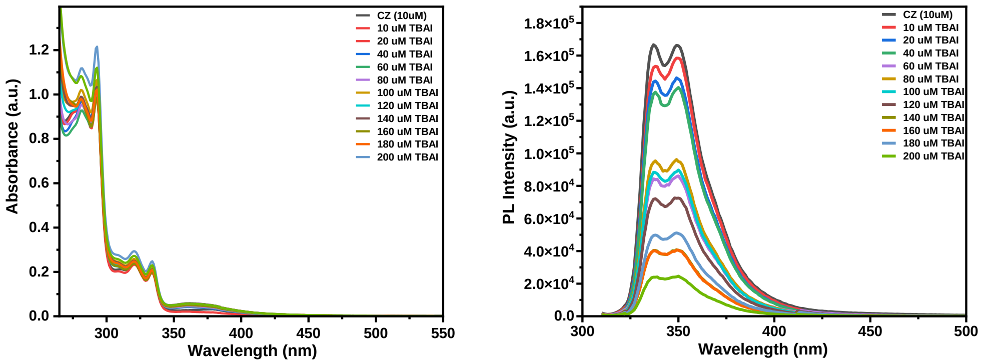

Figure S14 UV–vis and fluorescence spectra (λ_{ex}: 303 nm) of carbazole (10 μM) in $CHCl_3$ with TBAI concentration.

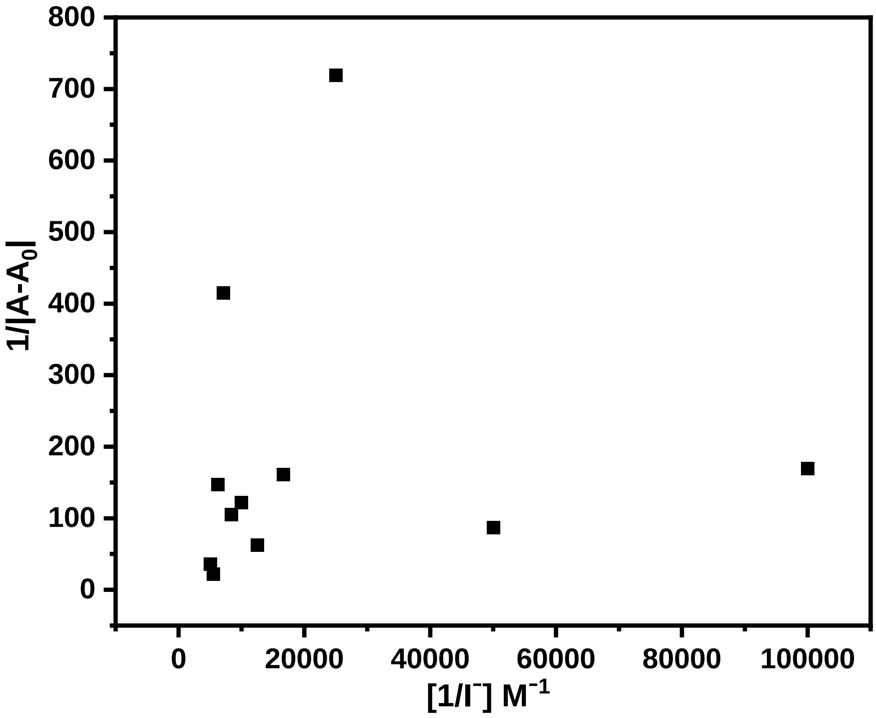

Figure S15 UV–is titration profile of carbazole with TBAI (non-Benesi–Hildebrand behavior).

6. Quantum chemical calculations

Quantum chemical calculations were performed with the ORCA 5.0.3 software[4]. The geometry optimization was performed by using B3LYP with the def2-SVP basis set.

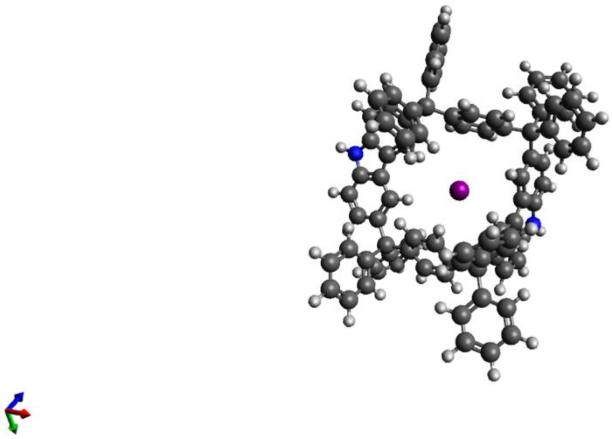

Figure S16 The computational results for the **PBG** and I^- complex

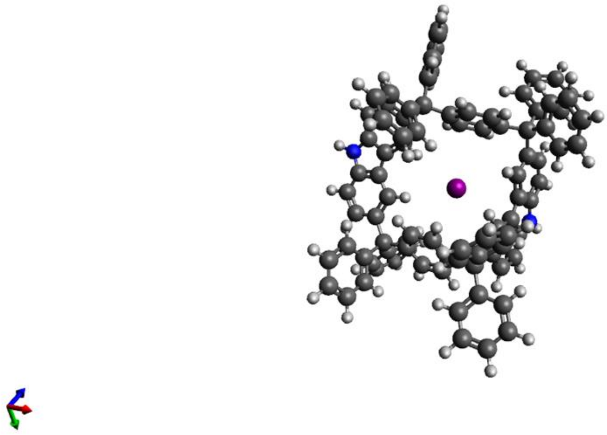

Figure S17 The computational results for the **WDG** and I^- complex

The Cartesian coordinates of the optimized complexes are as shown in the following text.

PBG and I^- complex

Energy: -3792.8875350

C	-1.97030	2.92710	-3.60310
C	-1.16030	1.85170	-3.60730
C	0.17200	1.95780	-3.47320
C	0.78860	3.15350	-3.44720
C	-0.01610	4.23030	-3.50480
C	-1.34900	4.12120	-3.58850
C	-3.49320	2.82830	-3.77300

C	2.30400	3.33400	-3.28810
C	-3.58760	2.57970	-5.28460
C	-4.19780	4.10310	-3.28370
C	3.15090	2.09100	-3.58580
C	2.90460	4.32180	-4.29540
C	-3.31860	3.56070	-6.16930
C	-3.35230	3.35020	-7.49420
C	-3.63270	2.12970	-7.97220
C	-3.85840	1.12680	-7.11200
C	-3.82120	1.35190	-5.78930
C	-5.14410	4.75080	-3.99170
C	-5.76200	5.84090	-3.51010
C	-5.46330	6.30140	-2.28700
C	-4.55360	5.65160	-1.54770
C	-3.94490	4.56380	-2.04320
C	4.23610	1.76880	-2.85400
C	4.99910	0.70460	-3.14830
C	4.71460	-0.05240	-4.21720
C	3.67770	0.28360	-4.99720
C	2.92400	1.35030	-4.68930
C	2.33830	4.55180	-5.49640
C	2.89500	5.38520	-6.38900
C	4.05710	5.99240	-6.10920
C	4.66140	5.74320	-4.93890
C	4.09420	4.90800	-4.05500
C	2.28450	4.73370	0.70310
C	2.30340	5.62370	-0.29120
C	2.33950	5.15090	-1.54780
C	2.36600	3.83010	-1.83620
C	2.30960	2.95710	-0.80750

C	2.29880	3.41800	0.44910
C	2.19000	3.51850	3.85900
C	2.23780	3.79680	2.55470
C	2.28390	2.81430	1.64520
C	2.32490	1.53060	2.02070
C	2.32920	1.21450	3.33520
C	2.22750	2.22630	4.22530
N	2.24660	4.91560	1.95760
C	-5.26370	-1.69850	-0.45540
C	-5.30860	-2.80380	0.29110
C	-4.14640	-3.26690	0.77810
C	-2.96810	-2.63030	0.59590
C	-2.96430	-1.49370	-0.13470
C	-4.11090	-1.04710	-0.66490
C	-6.33680	0.69210	-2.58140
C	-5.72370	-0.14260	-1.73990
C	-4.41100	-0.03650	-1.49450
C	-3.68070	0.92500	-2.07420
C	-4.25030	1.74590	-2.98310
C	-5.57890	1.61120	-3.20020
N	-6.19730	-1.12460	-1.09310
C	1.41160	-1.01570	2.93950
C	0.11850	-0.63890	2.98340
C	-0.84790	-1.31610	2.34230
C	-0.56650	-2.40060	1.59940
C	0.72580	-2.76990	1.54270
C	1.69050	-2.08400	2.17130
C	2.47190	-0.25820	3.75300
C	-1.65340	-3.29900	1.01210
C	2.30960	-0.47750	5.27050

C	3.89580	-0.79090	3.49190
C	-1.26810	-3.94420	-0.32630
C	-1.78810	-4.28860	2.17910
C	3.24160	0.00010	6.12240
C	3.15690	-0.17540	7.44990
C	2.12660	-0.85580	7.97020
C	1.19780	-1.36070	7.14660
C	1.29640	-1.17680	5.82040
C	4.86040	-0.07580	2.87900
C	6.09230	-0.57070	2.67940
C	6.40160	-1.80490	3.10030
C	5.46720	-2.53230	3.72740
C	4.24020	-2.02430	3.91920
C	-1.82070	-5.10770	-0.72340
C	-1.53480	-5.65610	-1.91440
C	-0.70480	-5.02990	-2.76050
C	-0.18430	-3.84540	-2.41010
C	-0.47840	-3.30920	-1.21560
C	-1.07100	-5.42840	2.23640
C	-1.14950	-6.26180	3.28550
C	-1.93130	-5.95590	4.33050
C	-2.61320	-4.80200	4.32100
C	-2.52510	-3.97900	3.26480
H	-1.58320	0.83940	-3.71240
H	0.75170	1.02400	-3.40650
H	0.41090	5.24570	-3.49020
H	-1.92130	5.05980	-3.66100
H	-3.05800	4.57210	-5.81730
H	-3.13710	4.17720	-8.19150
H	-3.65350	1.94780	-9.05950

H	-4.06010	0.11200	-7.49460
H	-3.97990	0.48790	-5.12450
H	-5.45010	4.39550	-4.98810
H	-6.52910	6.35330	-4.11500
H	-5.97450	7.19210	-1.88560
H	-4.31350	6.00530	-0.53090
H	-3.21540	4.03790	-1.40890
H	4.53750	2.38250	-1.99030
H	5.87360	0.46090	-2.52150
H	5.34450	-0.92140	-4.46960
H	3.45540	-0.30880	-5.90070
H	2.10280	1.61350	-5.37610
H	1.39940	4.05100	-5.78190
H	2.40440	5.56140	-7.36120
H	4.52200	6.67130	-6.84300
H	5.63090	6.21640	-4.70900
H	4.63090	4.70650	-3.11330
H	2.27820	6.70500	-0.08230
H	2.32500	5.90240	-2.35320
H	2.25980	1.87160	-0.98470
H	2.13480	4.32760	4.60490
H	2.39650	0.74840	1.24860
H	2.18840	2.03870	5.30740
H	2.22070	5.84800	2.42870
H	-6.25250	-3.35460	0.43030
H	-4.18800	-4.23790	1.29700
H	-2.02160	-0.97760	-0.37170
H	-7.41330	0.59440	-2.79350
H	-2.61930	0.98840	-1.80670
H	-6.08860	2.25980	-3.93090

H	-7.19380	-1.43840	-1.11660
H	-0.17310	0.22420	3.60560
H	-1.89380	-0.99620	2.47630
H	1.02060	-3.68080	0.99620
H	2.71510	-2.46530	2.05750
H	4.11130	0.55810	5.73550
H	3.93630	0.23510	8.11380
H	2.05110	-1.00900	9.05950
H	0.35510	-1.93820	7.56340
H	0.51280	-1.64070	5.20420
H	4.68270	0.95050	2.52630
H	6.85860	0.04120	2.17400
H	7.41330	-2.21290	2.94090
H	5.70920	-3.54650	4.08690
H	3.50210	-2.65520	4.44320
H	-2.53400	-5.63630	-0.07020
H	-1.99510	-6.61520	-2.20610
H	-0.47480	-5.47050	-3.74470
H	0.47300	-3.30480	-3.11200
H	-0.05640	-2.31760	-0.98430
H	-0.38570	-5.70370	1.41880
H	-0.55690	-7.19210	3.29810
H	-1.99090	-6.63190	5.19940
H	-3.23400	-4.52350	5.18910
H	-3.07560	-3.02490	3.30900
I	-1.74850	2.64050	1.26710

WDG and I^- complex

Energy: -3767.0500275

C	-4.44315	2.93211	-3.34744
C	-5.44156	1.97824	-3.46859

C	-5.11463	0.65685	-3.20986
C	-3.81789	0.29518	-2.80967
C	-2.84250	1.27601	-2.65817
C	-3.14046	2.60840	-2.94699
N	-5.89230	-0.47745	-3.31450
C	-5.10408	-1.58493	-3.08094
C	-3.81102	-1.16767	-2.72559
C	-5.41852	-2.93029	-3.18639
C	-4.41126	-3.85460	-2.95758
C	-3.11173	-3.47492	-2.59800
C	-2.82648	-2.11554	-2.46357
C	-0.43392	-3.45547	-4.35329
C	0.21843	-3.66638	-5.56253
C	0.14987	-4.89721	-6.20676
C	-0.56586	-5.94214	-5.64147
C	-2.32938	-7.99041	-3.82265
C	-3.10922	-8.65382	-2.88732
C	-3.56094	-7.98476	-1.75546
C	-3.23827	-6.64869	-1.54652
C	-1.14913	-4.49758	-3.78220
C	-1.20745	-5.73219	-4.42707
C	-2.01209	-6.65373	-3.61128
C	-2.46322	-5.97469	-2.47895
C	-1.96727	-4.51066	-2.47382
C	0.26343	-4.17950	-1.21312
C	0.98615	-4.07026	-0.03278
C	0.36393	-4.09819	1.20977
C	-1.02910	-4.14574	1.22079
C	-1.75151	-4.20632	0.04040
C	-1.12296	-4.27552	-1.20210

C	1.21130	-4.17835	2.49728
C	1.69277	-5.63421	2.66124
C	1.20026	-6.20160	3.83563
C	0.39177	-5.19437	4.54214
C	0.38095	-4.02309	3.78664
C	2.48636	-6.39168	1.81303
C	2.78620	-7.70541	2.15217
C	2.29307	-8.26664	3.32598
C	1.49400	-7.51698	4.17583
C	-0.28157	-5.27207	5.75579
C	-0.97171	-4.15923	6.21203
C	-0.98151	-2.98597	5.46393
C	-0.30360	-2.91123	4.25338
C	-3.29786	5.88092	-2.26691
C	-3.63329	7.18075	-2.62852
C	-3.18752	7.71890	-3.83039
C	-2.40073	6.95953	-4.68329
C	-0.61555	4.73231	-6.25393
C	0.11172	3.63654	-6.69444
C	0.19270	2.48954	-5.91176
C	-0.45878	2.41320	-4.68617
C	-2.51588	5.11127	-3.11573
C	-2.07054	5.65937	-4.31914
C	-1.25600	4.65776	-5.02315
C	-1.18565	3.50683	-4.23968
C	-2.00593	3.66237	-2.94195
C	-1.79213	3.64804	-0.40946
C	-1.07157	3.73053	0.77080
C	0.32187	3.69902	0.76646
C	0.94727	3.53811	-0.46449

C	0.22582	3.50358	-1.64999
C	-1.16171	3.58320	-1.65120
C	1.16527	3.93406	2.03760
C	0.33382	3.91538	3.33544
C	0.32927	5.16446	3.95397
C	1.12769	6.09504	3.13924
C	1.62951	5.40466	2.03683
C	-0.33854	2.85517	3.92412
C	-1.02006	3.05795	5.11785
C	-1.02581	4.30818	5.72868
C	-0.34770	5.37057	5.15049
C	1.40542	7.44371	3.32920
C	2.19780	8.10210	2.40105
C	2.70030	7.41791	1.29866
C	2.41631	6.07089	1.10946
C	3.57420	3.31352	2.68446
C	4.56992	2.40156	2.99138
C	4.28894	1.05178	2.83566
C	3.04951	0.62442	2.33642
C	2.08491	1.56329	1.98545
C	2.32155	2.91974	2.19167
N	5.05379	-0.05016	3.15224
C	4.30175	-1.18931	2.96165
C	3.05782	-0.83487	2.41856
C	4.59802	-2.50977	3.26796
C	3.61290	-3.46162	3.06651
C	2.35585	-3.13997	2.53385
C	2.10398	-1.81815	2.17639
H	-4.68923	3.95514	-3.58617
H	-6.43769	2.26217	-3.78320

H	-1.84554	0.99227	-2.34719
H	-6.79592	-0.50561	-3.73042
H	-6.41188	-3.25769	-3.46552
H	-4.64758	-4.90050	-3.07766
H	-1.83223	-1.78883	-2.18819
H	-0.33516	-2.49457	-3.87043
H	0.79434	-2.85721	-5.99122
H	0.66315	-5.04177	-7.14928
H	-0.61799	-6.90284	-6.13780
H	-1.97145	-8.50927	-4.70233
H	-3.36389	-9.69566	-3.03640
H	-4.16661	-8.50762	-1.02616
H	-3.60113	-6.15215	-0.65961
H	0.80781	-4.16577	-2.14259
H	2.05809	-3.95463	-0.10302
H	-1.57074	-4.13993	2.15435
H	-2.83064	-4.20517	0.09950
H	2.87348	-5.97431	0.89521
H	3.40610	-8.29828	1.49195
H	2.53137	-9.29324	3.57388
H	1.10604	-7.95303	5.08730
H	-0.26867	-6.18375	6.33899
H	-1.50240	-4.20140	7.15474
H	-1.52015	-2.12070	5.82849
H	-0.31889	-1.99151	3.68662
H	-3.65584	5.48671	-1.32813
H	-4.24425	7.77857	-1.96412
H	-3.45229	8.73401	-4.09864
H	-2.04738	7.37685	-5.61719
H	-0.67762	5.62872	-6.85776

H	0.62447	3.67614	-7.64744
H	0.77748	1.64225	-6.24391
H	-0.34988	1.51592	-4.09514
H	-2.87129	3.64038	-0.35156
H	-1.61519	3.82418	1.69852
H	2.02071	3.42868	-0.52002
H	0.77215	3.39115	-2.57164
H	-0.34173	1.87723	3.46482
H	-1.54933	2.23304	5.57725
H	-1.55899	4.45010	6.66018
H	-0.34684	6.34228	5.62716
H	1.01031	7.97529	4.18522
H	2.42388	9.15275	2.53203
H	3.31467	7.93987	0.57633
H	2.81056	5.55718	0.24513
H	3.77697	4.36095	2.84852
H	5.52544	2.73891	3.37175
H	1.14421	1.23014	1.57024
H	5.93145	-0.01890	3.61976
H	5.55726	-2.79124	3.68285
H	3.82731	-4.48163	3.34715
H	1.15960	-1.54474	1.72727
I	1.60349	-0.39870	-2.52762

7. References

(1) Zhang, G.; Zhi, X.; Zheng, S.; Huang, Q.; Xiang, J.; Xie, L.; Huang, W., *Dyes and Pigments* **2024,** *226*, 112129.

(2) Huang, G. B.; He, Z. F.; Cai, C. X.; Pan, F. F.; Yang, D. Q.; Rissanen, K.; Jiang, W., *Chemical Communications* **2015**, *51*, 15490.

(3) Yang, X. B.; Yang, B. X.; Ge, J. F.; Xu, Y. J.; Xu, Q. F.; Liang, J.; Lu, J.-M., *Organic*

Letters **2011,** *13*, 2710.
(4) Neese, F.; ORCA: An ab initio, density functional and semiempirical program package, V. 5.0.3, MPI für Kohlenforschung, Germany, **2022**.
